# Influence of diabetes mellitus on the pathological profile of aortic stenosis: a sex-based approach

**DOI:** 10.1186/s12933-023-02009-w

**Published:** 2023-10-17

**Authors:** Ernesto Martín-Núñez, Miriam Goñi-Olóriz, Lara Matilla, Mattie Garaikoetxea, Laura Mourino-Alvarez, Adela Navarro, Amaya Fernández-Celis, Ibai Tamayo, Alicia Gainza, Virginia Álvarez, Rafael Sádaba, María G. Barderas, Eva Jover, Natalia López-Andrés

**Affiliations:** 1 Cardiovascular Translational Research, Hospital Universitario de Navarra (HUN), Universidad Pública de Navarra (UPNA), C/Irunlarrea 3, 31008 Pamplona, Spain; 2 Department of Vascular Physiopathology, Hospital Nacional de Parapléjicos, Instituto de Investigación Sanitaria de Castilla-La Mancha (IDISCAM), Toledo, Spain; 3 Hospital Nacional de Paraplejicos, Servicio de Salud de Castilla-La Mancha (SESCAM), Toledo, Spain; 4 Research Methodology Unit, Navarrabiomed, Hospital Universitario de Navarra (HUN), Universidad Pública de Navarra (UPNA), IdiSNA, Pamplona, Spain

**Keywords:** Aortic stenosis, Sex-difference, Heart valve disease, Type 2 diabetes, Aortic valve disease

## Abstract

**Background:**

Diabetes mellitus (DM) accelerates the progression of aortic stenosis (AS), but how their underlying molecular mechanisms interact is not clear. Moreover, whether DM contributes to clinically relevant sex-differences in AS is unknown. In this work we aim to characterize the sex-specific profile of major pathological mechanisms fundamental to aortic valve (AV) degeneration in AS patients with or without concomitant DM.

**Methods:**

283 patients with severe AS undergoing surgical valve replacement (27.6% DM, 59.4% men) were recruited. Expression of pathological markers related to AS were thoroughly assessed in AVs and valve interstitial cells (VICs) according to sex and presence of DM. Complementary in vitro experiments in VICs in the presence of high-glucose levels (25 mM) for 24, 48 and 72 h were performed.

**Results:**

Oxidative stress and metabolic dysfunction markers were increased in AVs from diabetic AS patients compared to non-diabetic patients in both sexes. However, disbalanced oxidative stress and enhanced inflammation were more predominant in AVs from male AS diabetic patients. Osteogenic markers were exclusively increased in the AVs of diabetic women. Basal characterization of VICs confirmed that oxidative stress, inflammation, calcification, and metabolic alteration profiles were increased in diabetic VICs with sex-specific differences. VICs cultured in hyperglycemic-like conditions triggered inflammatory responses in men, whereas in women rapid and higher production of pro-osteogenic molecules.

**Conclusions:**

DM produces sex-specific pathological phenotypes in AV of AS patients. Importantly, women with diabetes are more prone to develop AV calcification. DM should be considered as a risk factor in AS especially in women.

**Supplementary Information:**

The online version contains supplementary material available at 10.1186/s12933-023-02009-w.

## Introduction

Aortic stenosis (AS) represents a major public health burden, being the leading cause of heart valve diseases in high-income countries. The progressive deterioration and dysfunction of the aortic valve (AV), eventually, leads to heart failure and death [1]. AS prevalence increases with age, accounting for 2–7% in subjects > 65 years [2–4]. Calcification, fibrosis, inflammation, and angiogenesis are some of the pathogenic hallmarks of AS [5]. No pharmacological treatments can delay or prevent AS. Either surgical or transcatheter AV replacement remain the only therapeutic options.

Diabetes mellitus (DM) is substantially more prevalent in patients with AS compared to general population, ranging between 11.4 and 31.6%, according to different studies [6–9]. Although the mechanisms linking AS and DM are not well understood, hyperglycemia is seemingly capable of triggering or accelerating AV inflammation, oxidative stress, and calcification [10–12]. In AS patients with concomitant DM, increased accumulation of advanced glycation end-products (AGEs) are associated with an accelerated deterioration of the AVs [13]. Similarly, valvular NF-κB expression, coagulation and calcification are increased in AS patients with DM compared to those without. All these processes are worsened in diabetic patients with poorly controlled glycaemia [13].

AS presents clear sex-specific differences in clinical presentation, patient management and pathophysiological mechanisms [14–16]. In patients with the same AS severity, men exhibit higher inflammation, apoptosis, calcification, lipid metabolic markers, angiogenesis and lymphangiogenesis than women [16–18]. Whether the DM might affect differently AS development and progression in men and women is still unknown. We aim to provide a thorough characterization of the general and sex-dependent effect of DM on the pathological features of AS.

## Methods

### Clinical cohort

This prospective and observational study included 283 patients with severe AS (AV area ≤ 1 cm^2^ and/or transaortic mean pressure gradient > 40 mmHg) referred to Hospital Universitario de Navarra for elective surgical AV replacement from June 2013 to November 2021. Moderate or severe concomitant valvular disease, malignant tumor, infective endocarditis and chronic inflammatory diseases (excluding DM) were elected as exclusion criteria. All patients were evaluated by transthoracic echocardiography and computed tomography. DM was diagnosed based on fasting serum glucose ≥ 126 mg/dL (7.0 mM) or on two-hour postprandial serum glucose > 200 mg/dL (11.1 mM) ( [19]). Venous blood was drawn on admission for surgery for the measurement of routine laboratory parameters.

AVs obtained from valve replacement surgery were cut, as much as possible, in three pieces. One third was used for VIC isolation and culture, another for protein and RNA extraction, and the last third for histological and immunohistochemistry analyses. All processed pieces were macroscopically similar aiming to minimize the heterogeneity of fibrocalcification within each AV, thus enhancing the accuracy of our quantifications Informed consent was obtained from each patient. The study protocol was approved by institutional human research committee (Comité Ético de Experimentación Clínica. Gobierno de Navarra, Departamento de Salud; Ethics numbers 17/2013 and PI2019/59) and it conforms to the ethical guidelines of the 1975 Declaration of Helsinki.

### Cell isolation and culture

Human VICs were isolated from 24 AVs (12 men and 12 women), as previously described [20]. In brief, AVs were minced and enzymatically digested into 2 mL of buffered-collagenase type 2 (240 U/mg of tissue) for 1 h. VICs were cultured in DMEM F-12 medium (Gibco) supplemented with 20% fetal bovine serum (FBS) (Gibco), 1% Penicillin-Streptomycin (Lonza), 5 µg/ml insulin (Sigma Aldrich) and 10 ng/ml of fibroblast growth factor (FGF-2) (Novus Biological) at 37 °C and 5% CO_2_ in a saturation humidified incubator (Panasonic). The VIC phenotype of isolated cells was confirmed at passage 1 by vimentin and alpha-smooth muscle actin (α-SMA) immunocytochemistry. Experiments were performed in serum-starvation conditions (1% FBS) in multiwell plates (Sarstedt). To model in vitro hyperglycemia-like conditions, VICs from both sexes were conditioned with high glucose (25 mM D-glucose) for 0, 24, 48 and 72 h. All experiments were carried out at passage 3–4, at least in 3 biological replicates (donors) *per* sex, with 6–8 technical replicates *per* condition.

### Histology and immunohistochemistry analysis

Histological determinations in whole AVs were performed in 5 μm-thick paraffin-embedded serial sections following the protocol of Leica BOND-Polymer Re-fine Detection automatic immunostainer (Leica). All solutions were filled into the bottle-Bond Open Container (Leica) and registered on computer using the Leica Biosystem program. The immunostaining program protocol include: fixative solution, bond wash solution, blocking with common immunohistochemistry blocker and incubation with the primary antibody for α-SMA (Sigma-Aldrich), CD68 (Abcam), CD45 (Santa Cruz Biotechnology), nitrotyrosine (Santa Cruz Biotechnology), malondialdehyde (MDA, Abcam), osteopontin (OPN, Santa Cruz Biotechnology), Runx2 (Sigma-Aldrich), insulin like growth factor binding protein 2 (IGFBP2, Santa Cruz Biotechnology) and fatty acid binding protein 4 (FABP4, Santa Cruz Biotechnology). After primary antibody incubation, slides were incubated with secondary poly-HRP-IgG. The signal was revealed by using DAB substrate. Incubation with no primary antibody was carried out in negative controls. Movat pentachrome staining, used to assess general histoanatomical features of the AVs, was performed following the manufacturer’s instructions (Abcam). Calcification was analyzed by alizarin red staining (2% in aqueous solution, pH: 4.1–4.3 with NH_4_OH, Sigma-Aldrich). Elastic stain kit was used to image elastic fibers according to the manufacturer’s instructions (Sigma-Aldrich). Histological preparations were imaged using bright field in an automated image analysis system, as appropriate (Nikon).

### ELISA

Leptin, adiponectin, FABP4 were measured in serum samples according to the manufacturer’s instructions (R&D Systems). Interleukin (IL)-6, C-C motif chemokine ligand 2 (CCL-2), CD44, myeloperoxidase (MPO), receptor for advanced glycosylation end-products (RAGE), endothelial nitric oxide synthase (eNOS), bone morphogenetic protein (BMP)-2, BMP-4, osteocalcin (OCN), periostin, adiponectin, leptin and IGFBP2 were measured in AVs protein lysates (N = 265) and cells supernatants according to the manufacturer’s instructions (R&D Systems). For explanted AV, equal micrograms of total protein (tissue lysates) were loaded and assayed by ELISA; for in vitro samples, equal volumes of cell supernatants were used and were thereafter normalized by the total micrograms of protein collected from the respective cell monolayers. Since the adiponectin/leptin ratio has been reported to be a more effective estimator of insulin resistance [21] and other metabolic risk factors [22, 23], we calculated it as an index of metabolic dysfunction in AVs and VICs.

### Western blot analyses

Aliquots of 5-7.5 µg of total proteins were prepared and electrophoresed from AV (N = 71) and VICs extracts on reducing SDS polyacrylamide gels (4–15% polyacrylamide, Mini-PROTEANTGX Stain-Free, BioRad) and transferred to Hybond-C Extra nitrocellulose membranes (BioRad). Primary antibodies used were superoxide dismutase 1 (SOD-1, Cell Signaling), fumarase, catalase and biglycan (all purchased from Santa Cruz Biotechnology). Secondary antibodies for mouse and rabbit were purchased from GE Healthcare. Stain free and β-actin were used as loading controls for blot normalization. Positive blots were detected with a chemiluminescence method (ECL, Amersham Biosciences) and images acquired with Chemidoc MP Imaging system (Bio-Rad). Semiquantitative analyses were performed by band densitometry using Image Lab software (Bio-Rad) and normalized data was expressed as arbitrary units (A.U.). All western blots were performed at least in triplicate for each experimental condition. Representative blots for markers assessed by western blot are shown in online supplemental material Figure S1.

### Quantitative PCR (qPCR)

Total RNA from VICs and AVs (N = 155) was extracted with Trizol Reagent (Canvas). First strand cDNA was synthesized according to the manufacturer’s instructions (Bio-Rad). Quantitative PCR analyses were performed using SYBR green PCR technology (Bio-Rad). Relative quantification was achieved with MyiQ (Bio-Rad) software according to the manufacturer’s instructions. Data were normalized to *18 S*, *HPRT*, *ACTB* and *GADPH* levels, and expressed as fold-change relative to men. All PCRs were performed at least in triplicate for each experimental condition. Primers sequences used have been previously reported [16].

### Statistical analyses

Demographic, clinical and biochemical data of the patients were summarized using percentages, means ± standard deviations (SD) or median [interquartile range], as appropriate. Data normality was assessed through Shapiro-Wilk’s and Kolmogorov-Smirnov’s tests. Differences between two groups were analysed by Mann–Whitney U test. Categorical variables were compared between groups using χ^2^ or Fisher exact tests.

Significant effects of DM, sex and their interaction (DM*sex) were assessed by gaussian generalized linear model with interaction. Briefly, variables were tested for normality of residuals and heteroscedasticity using White´s test. If homoscedasticity criteria was not fulfilled, variables were log transformed and reanalysed for assumption fulfilment. Same models were used to assess the magnitude of the interaction and reported as the Beta value of the magnitude, expressed as the numeric change in the analyte as a consequence of the concurrence of female sex and diabetes. For in vitro characterization of pathological markers, these were compared among groups according to sex and diabetes using ANOVA or Kruskal-Wallis accordingly followed by Dunn’s test in order to adjust the *p*-value for multiple comparisons, using non-diabetic male group as reference. A *p*-value < 0.05 was considered statistically significant. All analyses were performed using GraphPad Software Inc v.8.4.0 (GraphPad Software, San Diego, CA, USA) and R v. 4.3.1 (R Core Team (2022). R: A language and environment for statistical computing. R Foundation for Statistical Computing, Vienna, Austria.).

## Results

### Clinical data in AS cohort

Basal demographic and clinical characteristics of AS patients included in the study (median age: 71.5 [67–77] years, 59.4% men, 27.6% DM) are shown in Table 1. Diabetic AS patients presented higher body mass index, prevalence of arterial hypertension and renal insufficiency but less hypercholesterolemia compared to non-diabetic AS patients. These differences between diabetic and non-diabetic patients were maintained in men and women, with the exception of hypercholesterolemia and renal insufficiency, which were similar in women with or without DM. The higher use of angiotensin receptor blockers (ARBs) in the diabetic group was presented in women, whereas statin prescription was significantly higher in diabetic males. As expected, the intake of anti-diabetic drugs was exclusive to the diabetic group. Regarding echocardiographic parameters, there was a trend toward a greater AV area in female AS with DM as compared to non-diabetic. In diabetic male AS patients, end-systolic volume was significantly higher, whereas ejection fraction was lower compared to non-diabetic counterparts.

**Table 1:** Demographic and clinical data of AS patients

Variables	Total	Total		*p* value	Men		*p* value	Women		*p* value
		Non-diabetic	Diabetic		Non-Diabetic	Diabetic		Non-Diabetic	Diabetic	
*n* (%)	**283 (100)**	205 (72.4)	78 (27.6)		119 (42.1)	49 (17.3)		86 (30.4)	29 (10.2)	
Age, years	**71.5 [67–77]**	73 [66–78]	71 [67–77]	0.354	71 [65–77]	69 [63–74]	0.203	75 [67–79]	74.5 [71–78]	0.940
BMI, kg/m^2^	**29.4 [25.9–32.8]**	27.9 [25-31.4]	30.5 [27.4–33.4]	< 0.001	28.1 [25.7–31.6]	29.8 [27.6–32.7]	< 0.05	27.2 [24.6–31.3]	32.0 [27.1–36.1]	< 0.01
HTA, *n* (%)	**186 (65.7)**	123 (60)	63 (80.8)	< 0.01	76 (63.9)	39 (79.6)	< 0.0001	47 (54.7)	24 (82.8)	< 0.01
HCL, *n* (%)	**77 (27.2)**	69 (33.7)	8 (10.3)	< 0.0001	35 (29.4)	1 (2.04)	< 0.0001	34 (39.5)	7 (24.1)	0.179
Renal insufficiency, *n* (%)	**18 (6.4)**	7 (3.41)	11 (14.1)	< 0.01	2 (1.7)	7 (14.3)	< 0.01	5 (5.8)	4 (13.8)	0.227
**Drug medicines**										
ACEi, *n* (%)	**67 (23.7)**	45 (22)	22 (28.2)	0.277	26 (21.8)	13 (26.5)	0.549	19 (22.1)	9 (31)	0.330
ARB, *n* (%)	**67 (23.7)**	43 (21)	24 (30.8)	0.088	31 (26.1)	14 (28.6)	0.848	12 (14)	10 (34.5)	< 0.05
Diuretics, *n* (%)	**145 (51.2)**	100 (48.8)	45 (57.7)	0.187	56 (47.1)	26 (53.1)	0.502	44 (51.2)	19 (65.5)	0.202
β-blockers, *n* (%)	**82 (29)**	55 (26.8)	27 (34.6)	0.241	36 (30.3)	17 (34.7)	0.588	19 (22.1)	10 (34.5)	0.219
Statins, *n* (%)	**174 (61.5)**	118 (57.6)	56 (71.8)	< 0.05	78 (65.5)	42 (85.7)	< 0.01	40 (46.5)	14 (48.3)	> 0.999
Insulin, *n* (%)	**17 (6)**	0 (0)	17 (21.8)	< 0.0001	0 (0)	10 (20.4)	< 0.0001	0 (0)	7 (24.1)	< 0.0001
Metformin, *n* (%)	**50 (17.7)**	0 (0)	50 (64.1)	< 0.0001	0 (0)	33 (67.3)	< 0.0001	0 (0)	17 (58.6)	< 0.0001
Sulfonylureas, *n* (%)	**10 (3.5)**	0 (0)	10 (12.8)	< 0.0001	0 (0)	3 (6.12)	< 0.05	0 (0)	7 (24.1)	< 0.0001
DPP-4 inhibitors, *n* (%)	**10 (3.5)**	0 (0)	10 (12.8)	< 0.0001	0 (0)	8 (16.3)	< 0.0001	0 (0)	2 (6.9)	0.062
SGLT-2 inhibitors, *n* (%)	**3 (1.1)**	0 (0)	3 (3.8)	< 0.05	0 (0)	2 (4.1)	0.084	0 (0)	1 (3.4)	0.252
**Echocardiographic data**										
Maximal gradient, mmHg	**73 [66–89]**	76 [65-90.3]	73 [66-87.5]	0.315	74 [64.5–89.5]	71 [63.8–87]	0.210	79 [64.5–92.5]	76 [69–93]	0.948
Medium gradient, mmHg	**47 [41-56.8]**	48 [41–58]	47.5 [41-56.3]	0.754	47 [41–56]	46 [41–55]	0.920	50 [41.1–59]	49 [41–60]	0.823
Valvular area echocardiography, cm^2^	**0.79 [0.7–0.9]**	0.7 [0.6–0.9]	0.77 [0.66–0.8]	0.480	0.8 [0.7–0.9]	0.8 [0.7–0.8]	0.338	0.63 [0.58–0.79]	0.76 [0.6–0.8]	0.059
End-systolic volumen, cc	**44 [27.8–59.3]**	38 [23–55]	44 [23–64]	0.128	41 [27–54]	56 [41–70]	< 0.05	32 [18–58]	35 [20–52]	0.933
EF, %	**67 [57.8–75.3]**	66 [60-74.3]	64 [55–70]	0.054	67 [60–74]	58 [51–68]	< 0.001	65 [58–75]	67.5 [62–77]	0.187
**Biochemical data**										
Glucose, mg/dL	**110 [96–130]**	98 [91–107]	128 [110-159.5]	< 0.0001	97.5 [90.8–109]	125 [110–155]	< 0.0001	98 [91–106]	132 [109.3-172.3]	< 0.0001
eGFR, mL/min/1.73 m^2^	**78 [64–89]**	76.9 [64.2–85]	76.5 [57.3–88.6]	0.858	79.8 [66.5–89]	82.9 [66.3–89.8]	0.798	72 [61–79]	71 [54-83.3]	0.798
Creatinine, mg/dL	**0.89 [0.79–1.08]**	0.85 [0.75–0.99]	0.89 [0.8–1.1]	0.304	0.92 [0.82–1.08]	0.89 [0.80–1.11]	0.849	0.77 [0.71–0.86]	0.81 [0.71–0.97]	0.213
Total cholesterol, mg/dL	**164 [138–198]**	184.5 [157-210.3]	150 [127.5–177]	< 0.0001	173 [148.5-202.3]	142.5 [116.8-166.5]	< 0.0001	190 [171.3–221]	171.5 [141.5–201]	< 0.05
Triglycerides, mg/dL	**98.5 [75-133.8]**	91 [71.5–118]	109 [83.4-141.8]	< 0.01	88 [69–117]	104.5 [76-143.3]	0.066	94 [77.3–119]	123.5 [93-141.3]	< 0.05
HDL, mg/dL	**43 [35.8–53]**	48 [39-58.5]	37 [32-44.3]	< 0.0001	46 [39–55]	37 [32-44.3]	< 0.0001	51 [40–63]	44 [36.8–51.3]	< 0.05
LDL, mg/dL	**99 [74.5-122.5]**	113 [92.5–135]	88.5 [68.8-108.8]	< 0.0001	105 [84.8-130.3]	77 [62.3-103.5]	< 0.0001	119.5 [104.3-141.3]	100 [75-121.5]	< 0.05
Adiponectin, ng/mL	**27 [14-44.5]**	29 [16.5–48]	24 [8-35.8]	< 0.05	22 [14–34]	19 [7–33]	0.631	39 [23–58]	32.5 [11.5–56.8]	< 0.05
Leptin, ng/mL	**2.54 [1.42–4.7]**	2.46 [1.28–4.23]	3.34 [1.75–6.52]	< 0.01	1.65 [1.08–2.85]	2.14 [1.37–3.34]	0.589	4.61 [3.05–7.45]	7.26 [5.46–9.38]	0.151
FABP4, ng/mL	**49.95 [34.7–74.7]**	43.5 [33.6–64.7]	73.9 [48.7-112.3]	< 0.0001	39 [31-49.5]	66.5 [47.8–90.5]	< 0.0001	63.5 [40.8–82.3]	90 [45-130.3]	0.105

BMI, body mass index; HTA, arterial hypertension; HCL, hypercholesterolaemia; NYHA, New York Heart Association class; ACEi, Angiotensin-converting enzyme inhibitor; ARB, Angiotensin II receptor blocker; DPP-4: dipeptidyl peptidase 4; SGLT-2: sodium-glucose co-transporter-2; EF, Ejection fraction; eGFR, estimated glomerular filtration rate; HDL, High-density lipoprotein; LDL, Low-density lipoprotein; FABP4, Fatty-Acid binding protein 4

Serum glucose levels were higher in the AS diabetic group, both in men and women. The lipid profile in AS diabetic patients presented lower levels of total cholesterol, HDL and LDL but higher triglyceride levels in both sexes. Regarding serum metabolic markers, adiponectin showed lower levels in the AS diabetic group, but levels of leptin and FABP4 were significantly higher. However, these differences were not completely maintained when differentiating by sex. In AS men, only FABP4 levels were significantly higher in diabetic patients compared to their non-diabetic counterparts. In AS women, only serum adiponectin showed a significant difference, being lower in diabetic patients.

### Imaging and histological characterization of AVs in diabetic AS patients

**Fig. 1:**
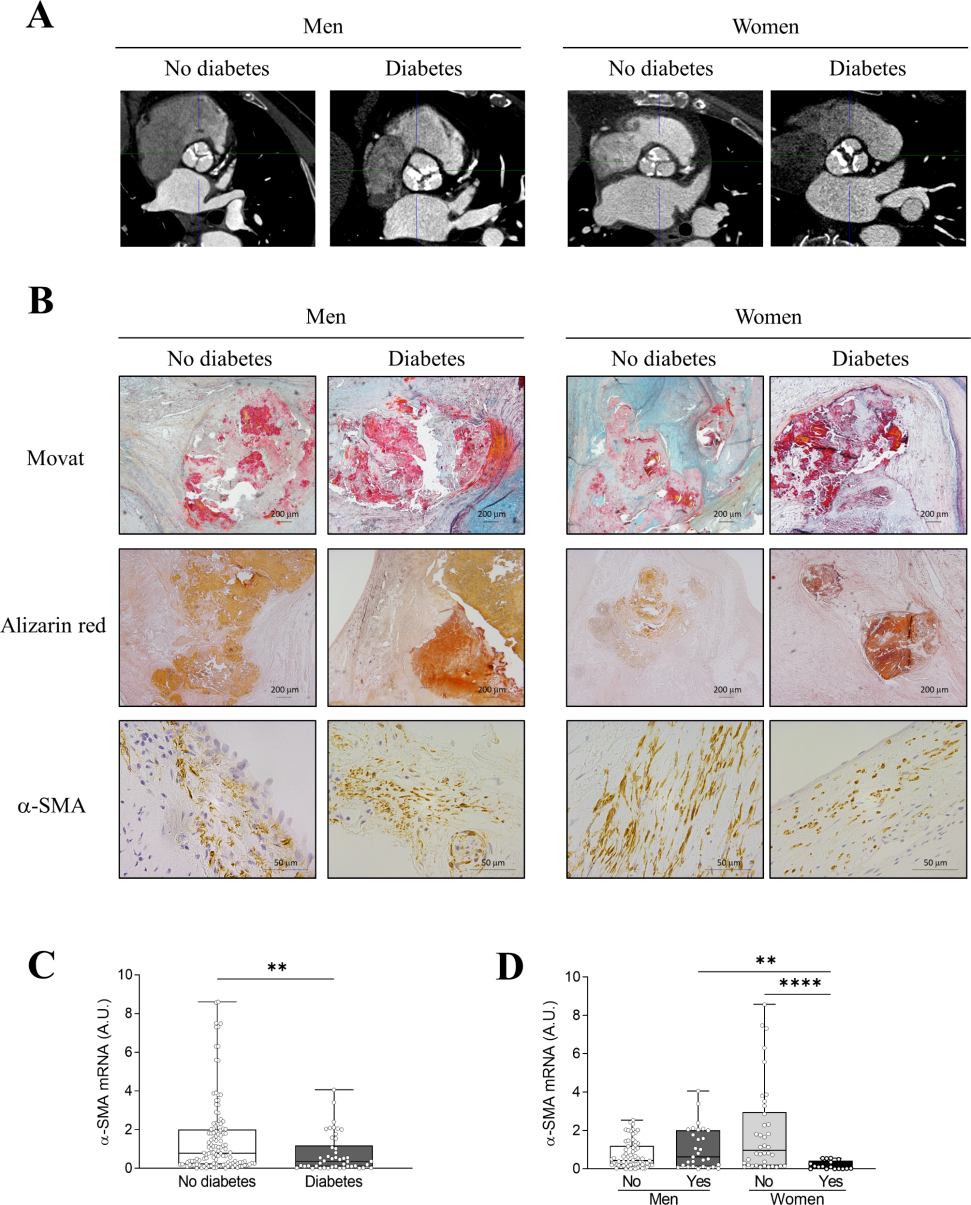
**Imaging and histological characterization of AVs from non-diabetic and diabetic AS patients according to sex**. Illustrative computed tomograms showing severe calcification of the aortic valve in the four groups of AS patients (**A**). Representative microphotographs of AVs from AS patients with Movat pentachrome staining (upper row), alizarin red staining (middle row) and α-SMA immunostaining (lower row) (**B**). mRNA levels of α-SMA in AVs of AS patients according only to the presence of diabetes (**C**) or to the presence of diabetes and sex (**D**). *N* = 155 AVs. **p < 0.01, ****p < 0.0001

All patients had significant AV calcification on computed tomography, although increased calcium content was shown in DM patients (Fig. 1A). Movat pentachrome staining revealed a greater presence of mineralized (bright yellow) and non-mineralized mature bone (bright red) in AVs from diabetic AS patients in both sexes, although in the case of females this difference was more evident (Fig. 1B). In agreement, alizarin red staining showed a greater presence of calcification in diabetic AS patients and, again, that was more evident in women (Fig. 1B).

Immunohistochemistry of α-SMA revealed a similar extent of VIC-to-myofibroblast activation in AVs of non-diabetic and diabetic men, whereas lesser α-SMA positive cells were observed in diabetic women (Fig. 1B). At the mRNA level, α-SMA expression was lower in diabetic patients that was only shown in women (Fig. 1C). Nevertheless, our immunohistochemistry observations in diabetic women were validated also at the transcript level (Fig. 1D). Interestingly, α-SMA mRNA expression levels in diabetic women were significantly lower than diabetic men (Fig. 1D). In agreement with all this, an analysis of the interaction between the presence of diabetes and sex showed that diabetes in women reduces α-SMA mRNA expression levels (Table S1 & Figure S2).

### Diabetes scales oxidative stress in stenotic AVs of both sexes but more predominantly in men

**Fig. 2:**
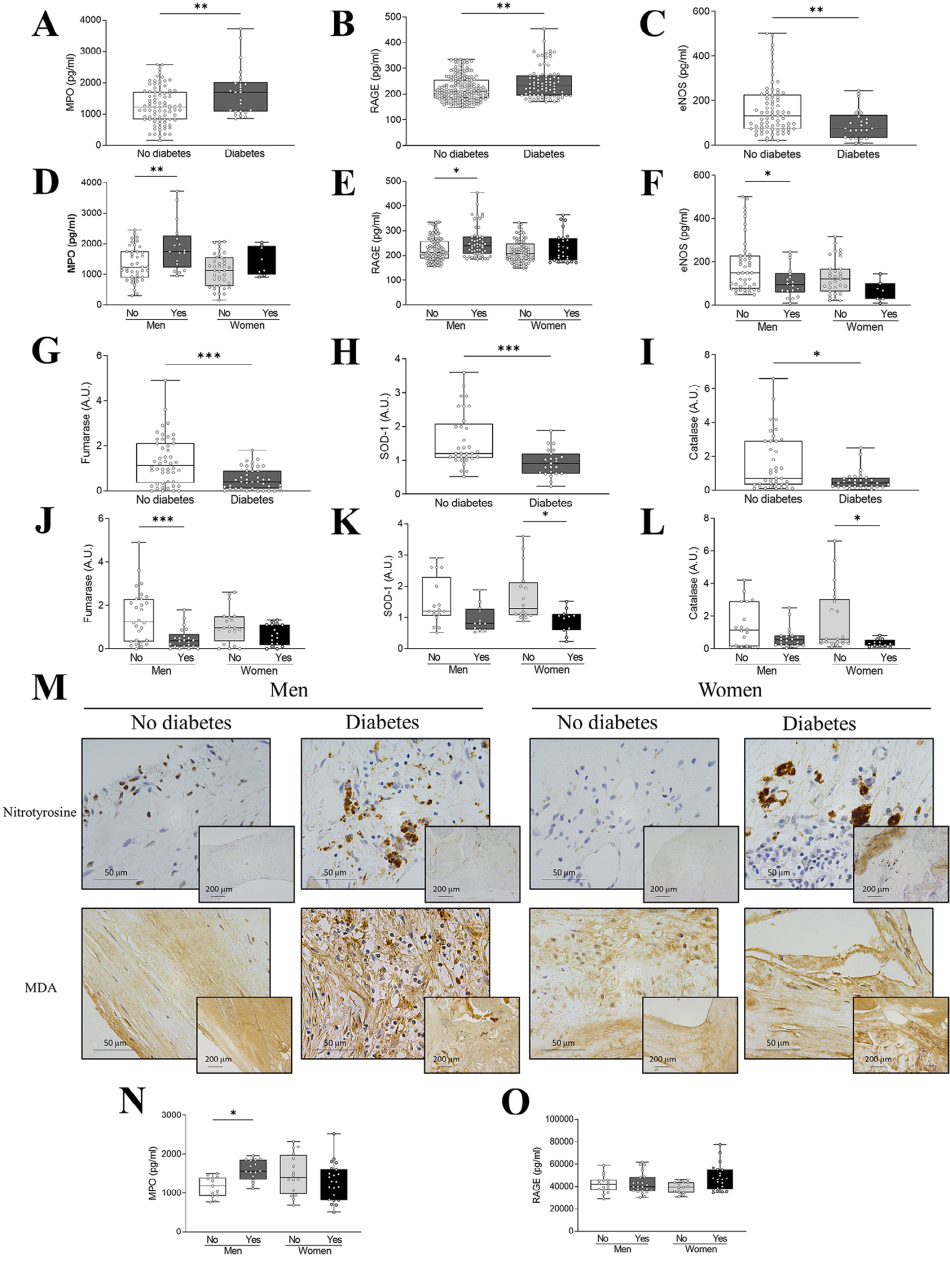
**Sex differences in oxidative stress markers in non-diabetic and diabetic AS patients**. MPO, RAGE and eNOS protein levels in tissue homogenates from AVs of AS patients according only to the presence of diabetes (**A**-**C**) or to the presence of diabetes and sex (**D**-**F**) measured by ELISA. Fumarase, SOD-1 and catalase protein expression in tissue homogenates from AVs of AS patients according only to the presence of diabetes (**G**-**I**) or to the presence of diabetes and sex (**J**-**L**) measured by western blot. Representative microphotographs of AV sections from non-diabetic and diabetic AS patients immunostained for nitrotyrosine (upper row) and MDA (lower row) according to sex (**M**). MPO (**N**) and RAGE (**O**) baseline protein levels in VICs derived from AVs of male and female non-diabetic and diabetic AS patients measured by ELISA. MPO, myeloperoxidase; RAGE, receptor for advanved glycation end-products; eNOS, endothelial nitric oxide synthase; SOD-1, superoxide dismutase 1; MDA, malondialdehyde. Protein expression measured by western blot was normalized to β-actin and stain free protein. Different sample sizes were assayed depending on the analytical methods used. *N* = 115–265 for ELISA, *N* = 56–87 AVs for western blot. In vitro experiments were performed in VICs derived from 3–4 donors *per* sex and condition with 6 technical replicates. *p < 0.05, **p < 0.01, ***p < 0.001

The cellular redox imbalance is intimately related to the development of DM and its complications. Accordingly, the pro-oxidative markers myeloperoxidase (MPO) and the surface receptor for AGEs (RAGE) were increased in AVs from diabetic AS patients (Fig. 2A-B). Such markers were enhanced in both sexes, but they only reached the statistical significance in diabetic men (Fig. 2D-E). In the opposite direction, AVs from diabetic patients expressed lower anti-oxidant markers eNOS, SOD-1, fumarase and catalase (Fig. 2C, G, H, I). Interestingly, eNOS and fumarase were significantly reduced only in diabetic men (Fig. 2F, J), whereas SOD-1 and catalase were significantly reduced only in diabetic women (Fig. 2K, L). Moreover, positive cells for nitrotyrosine and malondialdehyde (MDA) were more prevalent in AVs of diabetic patients (Fig. 2M). In addition, an interaction effect analysis showed that diabetes has no effect upon sex in the oxidative stress markers interrogated (Table S1 & Figure S2).

In vitro basal production of MPO levels was significantly increased only in diabetic male derived-VICs (Fig. 2N). No differences were found for the RAGE production among VICs from diabetic and non-diabetic AS patients from any sex (Fig. 2O). However, high-glucose conditioning of VICs increased MPO production in male-derived VICs after 72 h, while RAGE was increased after 48 h in both sexes (supplementry Figure S3A**&B**).

### Diabetes enhances inflammation in AVs of male AS patients

**Fig. 3:**
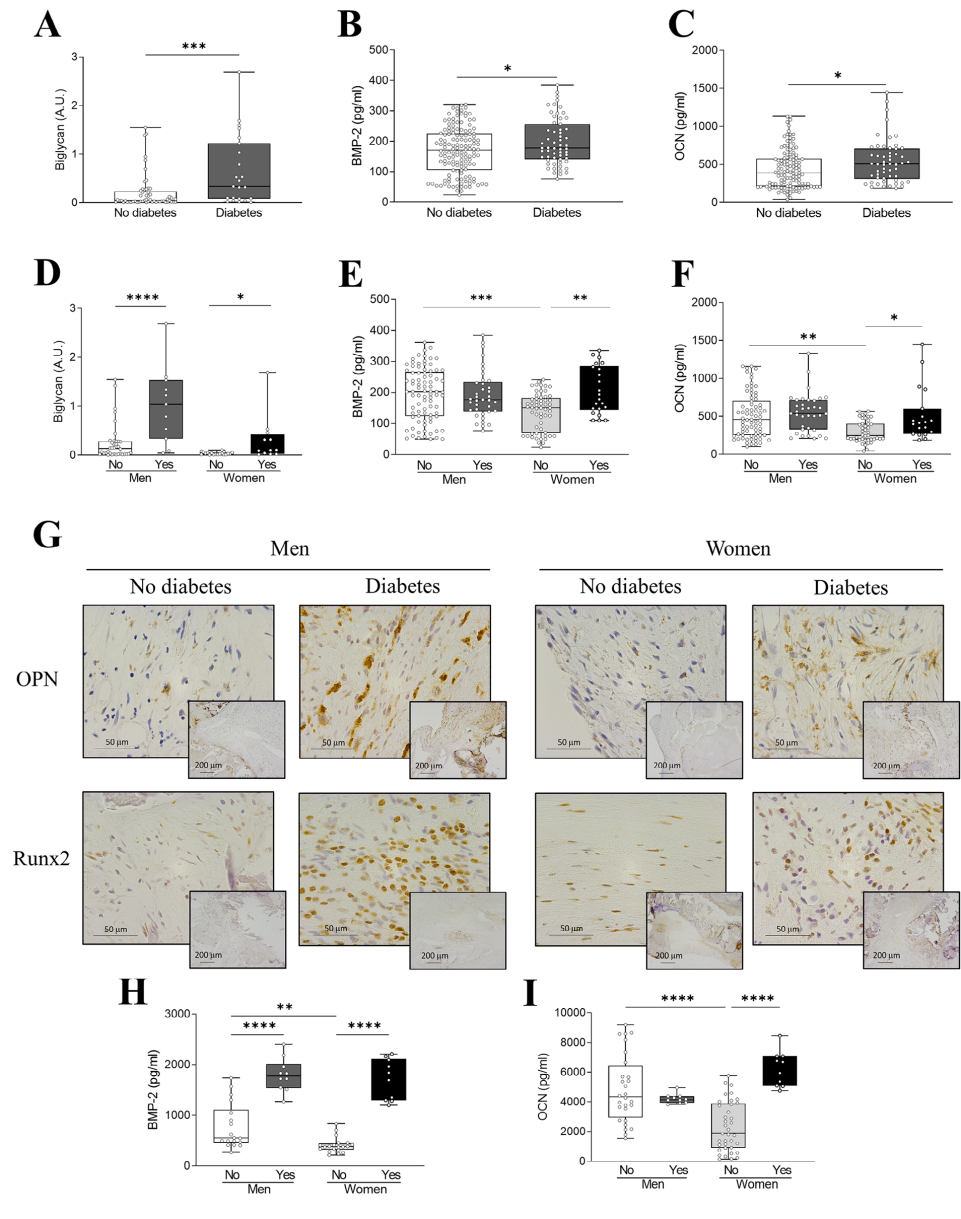
**Sex differences in inflammatory markers in non-diabetic and diabetic AS patients**. IL-6, CCL-2 and CD44 protein levels in tissue homogenates from AVs of AS patients according only to the presence of diabetes (**A**-**C**) or to the presence of diabetes and sex (**D**-**F**) measured by ELISA. Representative microphotographs of AV sections from non-diabetic and diabetic AS patients immunostained for CD68 (upper row) and CD45 (lower row) according to sex (**G**). IL-6 (**H**) and CCL-2 (**I**) baseline protein levels in VICs derived from AVs of male and female non-diabetic and diabetic AS patients measured by ELISA. IL, interleukin; CCL, C-C motif chemokine ligand; CD, cluster differentiation. *N* = 95–206 AVs analyzed by ELISA. In vitro experiments were performed in VICs derived from 3–4 donors *per* sex and condition with 6 technical replicates. *p < 0.05, **p < 0.01, ****p < 0.001

AVs from diabetic patients presented higher expression of inflammatory markers IL-6 and CD44 compared to non-diabetic ones (Fig. 3A**&C**). Such enhanced expression was only observed in AVs from diabetic men (Fig. 3D**&F**). Interestingly, diabetic men showed higher levels of IL-6 than diabetic women (Fig. 3H). No differences were reported for CCL-2 expression between AVs from diabetic and non-diabetic patients (Fig. 3B**&E**). After sex classification, diabetic men presented significantly higher expression of this chemokine (Fig. 3E). Besides, CD68 and CD45 positive cells were more prevalent in AVs of diabetic men (Fig. 3G). Likewise, an analysis of the interaction effect showed that the presence of diabetes in men increases the levels of inflammatory markers (Table S1 & Figure S2).

In vitro cultured non-diabetic male VICs produced higher basal levels of IL-6 and CCL-2 than women’s. VICs derived from diabetic patients produce significantly higher levels both in men and women than their non-diabetic counterparts. No differences were found between sexes in diabetic VICs (Fig. 3H-I).

In vitro high glucose increased IL-6 levels at 48 h in male and female non-diabetic VICs. Interestingly, the percentage increase in IL-6 levels at 72 h was more pronounced in men (254%) than in women (51.8%). Also, CCL-2 was increased after 72 h of treatment only in male VICs (supplementry Figure S3C**&D**).

### Calcific cues are over-represented in AVs of female diabetic AS patients

**Fig. 4:**
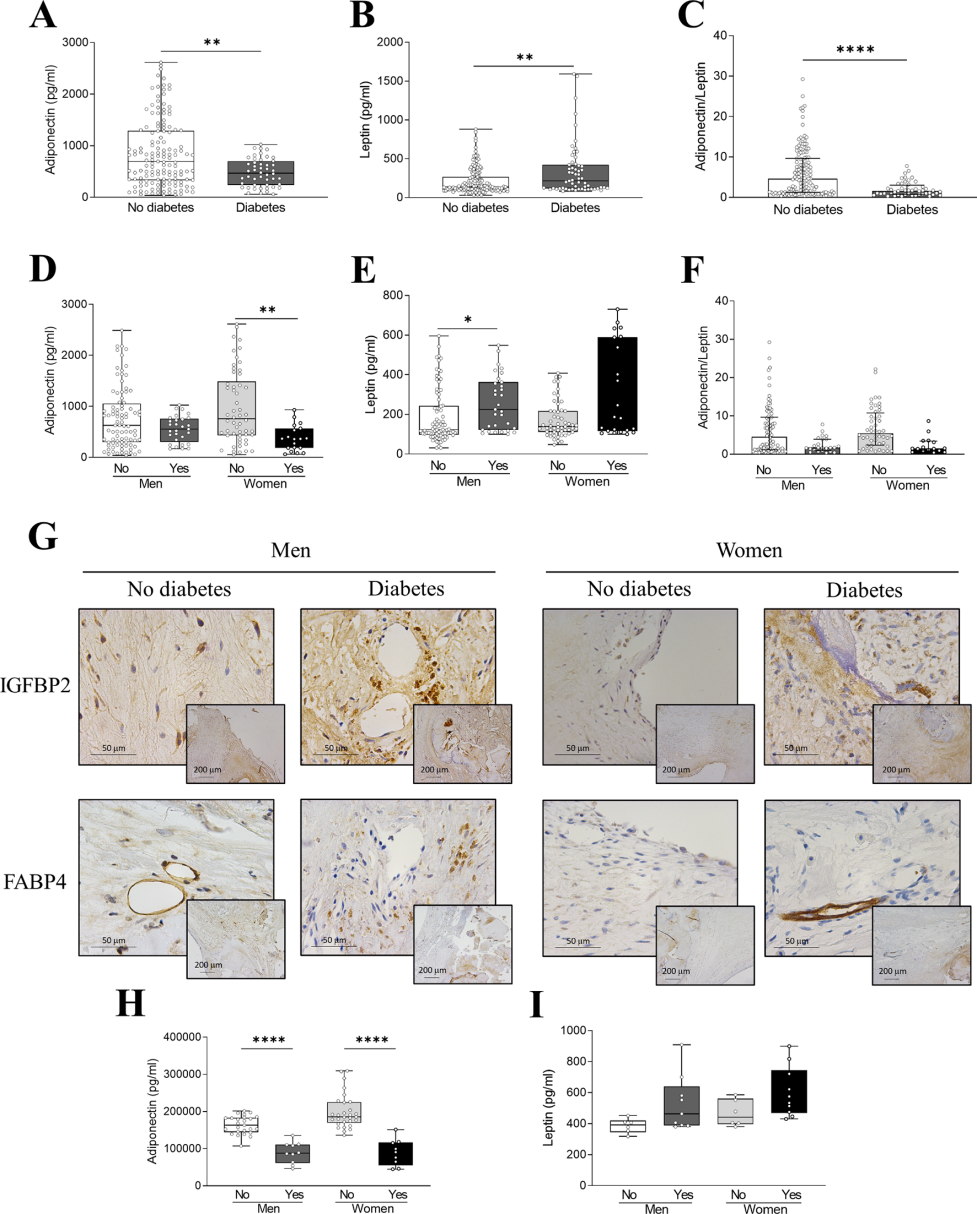
**Sex differences in calcification markers in non-diabetic and diabetic AS patients**. Biglycan, BMP-2 and OCN protein levels in tissue homogenates from AVs of AS patients according only to the presence of diabetes (**A**-**C**) or to the presence of diabetes and sex (**D**-**F**) measured by western blot or ELISA. Representative microphotographs of AV sections from non-diabetic and diabetic AS patients immunostained for OPN (upper row) and Runx2 (lower row) according to sex (**G**). BMP-2 (**H**) and OCN (**I**) baseline protein levels in VICs derived from AVs of male and female non-diabetic and diabetic AS patients measured by ELISA. BMP, bone morphogenetic protein; OCN, osteocalcin; OPN, osteopontin. Protein expression measured by western blot was normalized to β-actin and stain free protein. Different sample sizes were assayed depending on the analytical methods used. *N* = 179–201 AVs for ELISA and *N* = 44 AVs for western blot. In vitro experiments were performed in VICs derived from 3–4 donors *per* sex and condition with 6 technical replicates. *p < 0.05, **p < 0.01, ***p < 0.001, ****p < 0.001

Increased expression of biglycan, BMP-2 and osteocalcin was observed in diabetic AS patients. Interestingly, BMP-2 and osteocalcin markers were increased only in AVs from diabetic women, even reaching similar levels to men’s (Fig. 4B-C-E-F). Accordingly, interaction effect analysis showed that diabetes in women increases BMP-2 and osteocalcin levels (Table S1 & Figure S2). Similarly, basal expression of BMP-2 and osteocalcin in non-diabetic male VICs were significantly higher than in female counterparts, but these sex differences were not reported upon high glucose conditioning (Fig. 4H-I). Immunohistochemistry revealed a higher presence of osteopontin and Runx2 positive cells in AVs sections of both sexes (Fig. 4G).

In vitro high glucose increased BMP-4 and periostin at 48 and 72 h, respectively, in male VICs. However, female VICs showed significant increases in the same markers as after only 24 h. In addition, the percentage increase at 72 h of these markers was much more pronounced in women than men (11.5% in men vs. 63.3% in women for BMP-4; 37.3% in men vs. 114.9% in women for periostin) (supplementry Figure S3E**&F**).

### Diabetes produces an imbalance in metabolic markers in stenotic AVs in both sexes

**Fig. 5:**
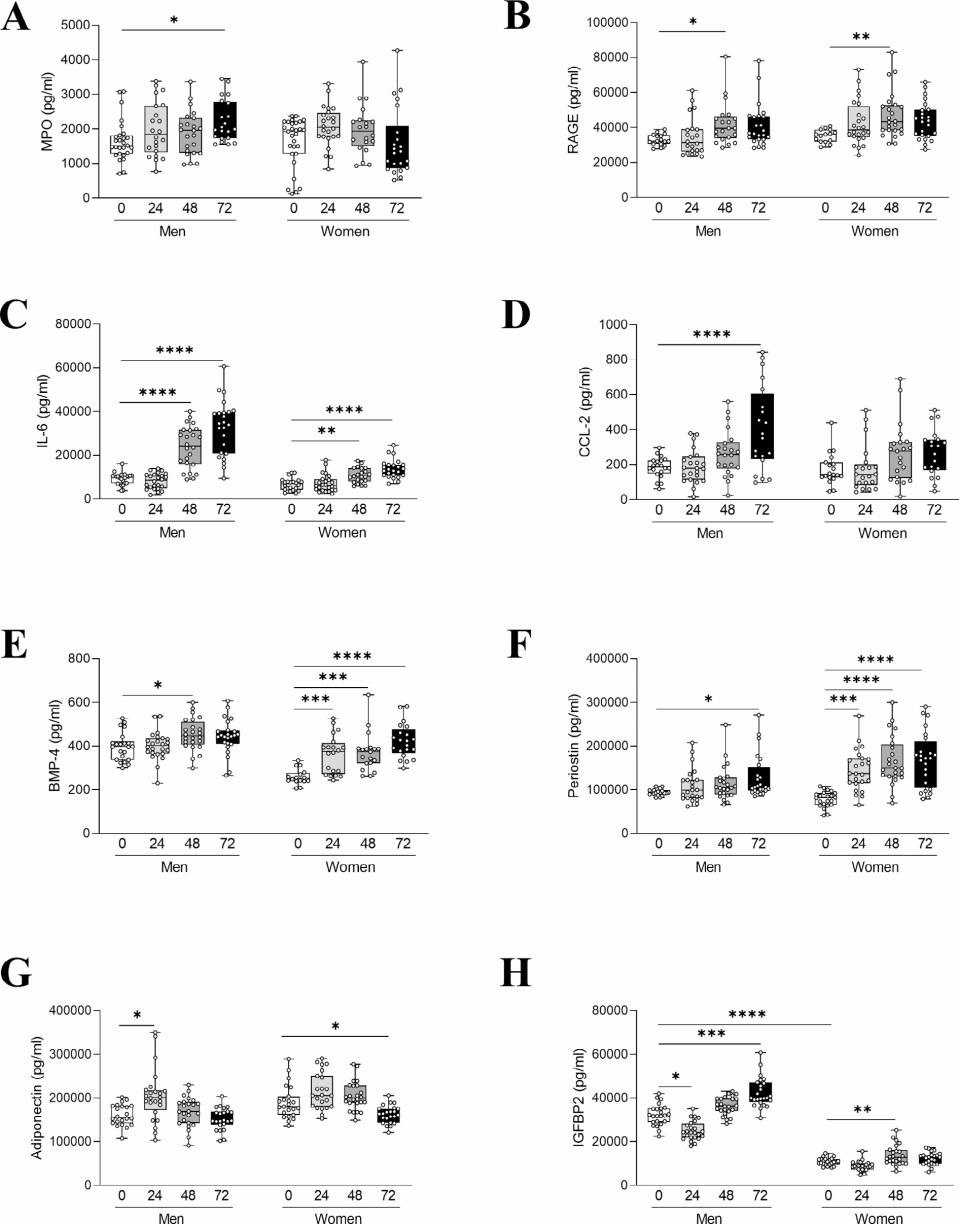
**Sex differences in metabolic markers in non-diabetic and diabetic AS patients**. Adiponectin, leptin and adiponectin-to-leptin ratio of protein levels in tissue homogenates from AVs of AS patients according only to the presence of diabetes (**A**-**C**) or to the presence of diabetes and sex (**D**-**F**) measured by ELISA. Representative microphotographs of AV sections from non-diabetic and diabetic AS patients immunostained for IGFBP2 (upper row) and FABP4 (lower row) according to sex (**G**). Adiponectin (**H**) and leptin (**I**) baseline protein levels in VICs derived from AVs of male and female non-diabetic and diabetic AS patients measured by ELISA. IGFBP, insulin like growth factor binding protein; FABP, fatty-acid binding protein. *N* = 192 AVs analyzed by ELISA. In vitro experiments were performed in VICs derived from 3–4 donors *per* sex and condition with 6 technical replicates. *p < 0.05, **p < 0.01, ****p < 0.001

The production of adipokines in AVs of diabetic patients showed changes with respect to non-diabetic AS patients (Fig. 5A-F). Specifically, adiponectin levels were reduced in the diabetic condition (Fig. 5A), and that remained significant in the female group after sex classification (Fig. 5D**)**. Leptin levels were increased in diabetic patients (Fig. 5B) and that only remained significant in male (Fig. 5E) after sex classification. Although diabetes in women reduces adiponectin levels, no effect was observed in the interaction between the presence of diabetes and sex for leptin levels (Table S1 & Figure S2). The adiponectin/leptin ratio was decreased in AS patients with concomitant DM of both sexes (Fig. 5F). VICs derived from diabetic AS patients showed significantly lower basal levels of adiponectin but not of leptin in both sexes (Fig. 5H-I). Immunohistochemistry of IGFBP2 and FABP4 revealed a higher presence of these metabolic markers in AVs sections of male and female diabetic patients (Fig. 5G).

Exposition to high glucose in VICs showed that adiponectin production was increased after 24 h (only significant in male) just to be followed by a decreased at 72 h (levels similar to baseline in men or lower in the case of women). In the case of IGFBP2 production, baseline levels were significantly higher in men than in women and an increase in its production was observed in both sexes after 48 (in women) or 72 h (in men) exposure (supplementry Figure S3G**&H**).

## Discussion

Our results demonstrate that AVs of AS patients with DM exhibit a greater degree of oxidative stress, inflammation, calcification and aberrant metabolic profiles. But importantly, we also show for the first time an interaction between diabetes and sex on the pathological features of AS in the AVs. Thus, inflammation appeared as a process increased by diabetes in males, whereas VIC to myofibroblast activation and calcification were processes increased in females by the presence of diabetes. The alteration of oxidative stress and metabolic markers due to DM occurred independently of the sex. Basal characterization of VICs showed sex-specific profiles in oxidative stress, inflammation, calcification and metabolic alterations in diabetic vs. non-diabetic-derived cells. Nevertheless, high glucose triggered a more pronounced inflammation in non-diabetic VICs from men, whereas inducing a rapid and higher production of pro-calcifying markers in women. The hyperglycemic stress of the diabetic condition might have early sex-dependent effects on the VIC reaching similar levels in both sexes upon chronification. Consequently, these findings could be clinically relevant to the management and outcomes of AS with or without DM in a sex-dependent manner.

Hyperglycemia and metabolic disorders are associated to increased production of oxidative stress, [25] chronic inflammation and fibrosis. A high oxidative stress can induce AV fibrosis and osteogenesis through increased expression of NADPH oxidase 2 (NOX2) and BMP-2 [12, 26]. Our results showed an impaired balance between pro- and anti-oxidant systems in the diabetic stenotic AV pointing to increased oxidative stress. Expression of MPO was increased in AVs only in diabetic men. Interestingly, eNOS and fumarase levels decreased in diabetic men, and SOD-1 and catalase in diabetic women. Moreover, nitrotyrosine or MDA showed an increase presence in AVs from both diabetic men and women. Despite these observed differences, interaction effect analyses showed that diabetes had no effect on sex for these markers. Chronic hyperglycemia accelerates AGEs formation which binding to the cell surface RAGE enhances multiple cellular processes such as oxidative stress, inflammation and coagulation activation [27]. Indeed, concomitant DM with AS was associated with increased valvular AGEs and RAGE compared to non-diabetics AS patients [13]. Accumulation of AGEs in AVs induces endothelial mesenchymal transition, NF-κB overexpression and promotes AV calcification [13, 28–32]. We also observed that valvular RAGE levels are augmented only in diabetic men. Interestingly, baseline RAGE levels in VICs of diabetic patients did not differ significantly from those of non-diabetic patients. This could suggest that the increased presence of this receptor observed in diabetic AVs could be importantly due to the VECs present in the fibrosa and ventricularis layers, which are the first barrier in contact with the accumulated AGEs in the hyperglycemic blood. Nevertheless, we cannot rule out that other cell types (e.g. circulating cells recruited) may contribute to the phenotype of the diabetic stenosis AV. Regardless of sex, VICs exposed to hyperglycemic-like conditions showed increased RAGEs indicating that these cells are sensitive to the AGE/RAGE accumulation.

Inflammation plays a central role in initiation and progress of AS pathogenesis [33, 34]. AVs from AS patients with concomitant DM present increased C-reactive protein-positive areas and transcript levels which correlates with coagulation factors [10]. Likewise, diabetic AS patients present enhanced NF-κB valvular expression [35]. In agreement with this, our results showed that diabetes has an effect on inflammatory markers in AVs according to sex, with an upregulation of inflammatory processes in diabetic men. In vitro, hyperglycemic-like conditions upregulated inflammation only in male VICs, except for IL-6 which is also upregulated in female-derived VICs but to a lower extent. Interestingly, basal production of IL-6 and CCL-2 was increased in diabetic-derived VICs in both sexes compared to non-diabetic cells, suggesting a ‘pro-inflammatory memory’ in the diabetic VIC.

Macrocalcification is the ultimate feature of degenerated valves in AS. DM can participate in the progression of AV calcification, since hyperglycemia, insulin resistance and dyslipidemia enhance these pathological processes [36]. Histological analyses of AVs in AS patients revealed that concomitant DM is associated with higher calcification and early osteogenic markers [37]. Increased expression of NF-κB in AV of diabetic AS patients is directly associated to augmented levels of BMP-2 [35]. Our results show that diabetic patients with AS in the whole cohort have a greater degree of valvular calcific degeneration. Interestingly, only in diabetic women these characteristics were significantly exacerbated together with less activated valve cells. Also, interaction effect analysis showed that the presence of diabetes in women augmented the levels of calcification markers. As previously shown, AVs and VICs of male AS patients presented increased expression of bone markers compared to women [16, 24]. In the current study considering the particular effect of diabetes, we observed that osteogenic markers in AVs or in VICs of non-diabetic AS patients showed higher levels in men than in women. However, the increase observed in diabetic women in such markers equalled the values in men. Moreover, high glucose experiments in VICs showed that the upregulation of osteogenic molecules occurs rapidly and to a greater extent in female AV-derived cells.

The influence of adipocytokines in AS has not been widely studied. Changes in adiponectin and leptin levels are associated to the degree of severity of AS [38, 39]and to an increased risk of metabolic syndrome or DM [40]. A diminished adiponectin/leptin ratio is related to increased insulin resistance, oxidative stress and inflammation [21, 23]. In our cohort of AS, these adipokines were overall altered in diabetic AVs with lower levels of adiponectin/leptin ratio. Interestingly, adiponectin was significantly diminished only in diabetic women, while leptin was increased in diabetic men. In vitro, high glucose diminished adiponectin only in VICs from women. Recently, we have demonstrated that FABP4 is expressed in AVs from AS patients and associated to inflammatory, apoptotic and calcification markers in men [18]. Now we showed that FABP4 and IGFBP2 were enhanced in diabetic AVs from both sexes. Accordingly, IGFBP2 levels were overall increased in high glucose-conditioned VICs. These results indicate that DM is generally related to an increased metabolic imbalance in AS.

We acknowledge limitations in our study. First, a small number of diabetic AS female patients were recruited, likely limiting our statistical analyses and explaining some of the discrepancies observed for clinical data between sexes (e.g. renal insufficiency or drug usage). Second, the average age of our study group (72, [67–77] years) were relatively younger than the data provided by the different studies on degenerative AS, because it is composed of patients undergoing elective surgical aortic valve replacement. Second, long-term glycaemic control measures were only available for very few diabetic patients, limiting any further analyses to explore whether poorly controlled glycaemia has a potential sex-dependent impact [13, 35].

## Conclusion

We provide new evidence on the influence of diabetes concomitant to AS on sex with respect to the pathological hallmarks of AV degeneration. Thus, the presence of diabetes leads to an overall imbalance in oxidative stress and metabolic processes regardless of sex, while is shown to have an effect on inflammation with an increase in pro-inflammatory markers exclusively in AV of men. More importantly, our results indicate that diabetes in women with AS would lead to a calcifying phenotype of the AV. Since stenotic AVs in women are usually characterized by a fibrotic phenotype, this finding would indicate that diabetes might contribute to a more accelerated AV Such new hypothesis would need of future studies to test it, as it might have potential clinical relevance in the reconsideration of the management and treatment of this type of patient.

### Electronic supplementary material

Below is the link to the electronic supplementary material.

Additional file 1: Figure S1. Representative western blot for oxidative stress and calcification markers in AVs of non-diabetic and diabetic AS patients

Additional file 2: Table S1. Effect interaction analysis of the presence of DM and sex in AS cohort

Additional file 3: Figure S2. Effect interaction plots between the presence of diabetes and sex for pathological markers of AS in AVs from AS patients

Additional file 4: Figure S3. Expression of oxidative stress, calcification, inflammatory and metabolic markers in non-diabetic VICs exposed to high-glucose levels

## Data Availability

The datasets used and/or analysed during the current study are available from the corresponding author on reasonable request.

## Abbreviations

AS: Aortic stenosis
AV: Aortic valve
α-SMA: Alpha-smooth muscle actin
BMP: Bone morphogenetic protein
CCL-2: C-C motif chemokine ligand
DM: Diabetes mellitus
eNOS: Endothelial nitric oxide synthase
FABP4: Fatty acid binding protein 4
IGFBP2: Insulin like growth factor binding protein 2
MPO: Myeloperoxidase
OCN: Osteocalcin
OPN: Osteopontin
RAGE: Receptor for advanced glycation end-products
SOD-1: Superoxide dismutase 1
VIC: Valve interstitial cell
