# Supplementary material for: Influence of diabetes mellitus on the pathological profile of aortic stenosis: a sex-based approach
Source: Cardiovasc Diabetol. 2023 Oct 17;22:280. doi: 10.1186/s12933-023-02009-w (PMC10583330; doi:10.1186/s12933-023-02009-w)
Supplement: Supplementary file 1 — Additional file 1: Figure S1. Representative western blot for oxidative stress and calcification markers in AVs of non-diabetic and diabetic AS patients [file 12933_2023_2009_MOESM1_ESM.pptx]

## Slide 1
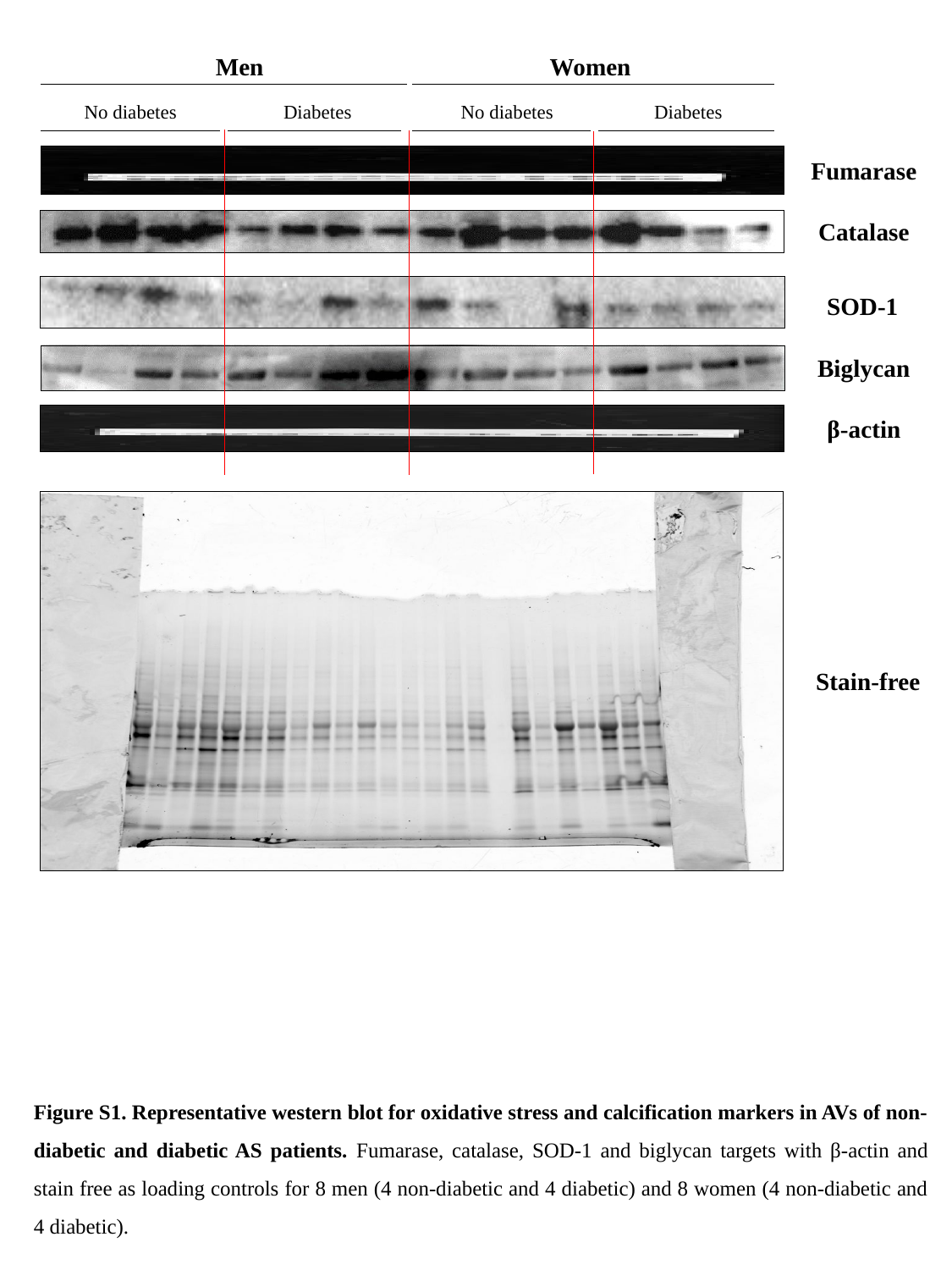

Men
Women
No diabetes
No diabetes
Diabetes
Diabetes
Fumarase
Catalase
SOD-1
Biglycan
β-actin
Stain-free
Figure S1. Representative western blot for oxidative stress and calcification markers in AVs of non-diabetic and diabetic AS patients. Fumarase, catalase, SOD-1 and biglycan targets with β-actin and stain free as loading controls for 8 men (4 non-diabetic and 4 diabetic) and 8 women (4 non-diabetic and 4 diabetic).
