# Supplementary material for: Influence of diabetes mellitus on the pathological profile of aortic stenosis: a sex-based approach
Source: Cardiovasc Diabetol. 2023 Oct 17;22:280. doi: 10.1186/s12933-023-02009-w (PMC10583330; doi:10.1186/s12933-023-02009-w)
Supplement: Supplementary file 2 — Additional file 2: Table S1. Effect interaction analysis of the presence of DM and sex in AS cohort [file 12933_2023_2009_MOESM2_ESM.docx]

**Table S1. Effect interaction analysis of the presence of DM and sex in AS cohort.**

| **Variable** | **Beta (DM*Sex)** | **p-value (DM*Sex)** | **p-value White** |
| --- | --- | --- | --- |
| mpo | -415.32 (-1083.4 ; 252.76) | 0.223 | 0.633 |
| rage | -10.59 (-39 ; 17.82) | 0.465 | 0.224 |
| enos | -22.83 (-126.85 ; 81.19) | 0.667 | 0.361 |
| fumarasa | 0.61 (-0.13 ; 1.34) | 0.108 | 0.050 |
| sod1 | -0.27 (-0.99 ; 0.45) | 0.459 | 0.286 |
| catalasa | -0.6 (-1.84 ; 0.63) | 0.338 | 0.063 |
| il_6 | -27.12 (-47.97 ; -6.28) | 0.011* | 0.284 |
| ccl_2 | -51.58 (-79.47 ; -23.69) | 0.000*** | *0.016* |
| cd44s | -1221.32 (-1857.52 ; -585.11) | 0.000*** | *0.000* |
| biglycan | -0.49 (-0.91 ; -0.06) | 0.024* | 0.099 |
| bmp_2 | 70.05 (22.21 ; 117.89) | 0.004** | 0.112 |
| ocn | 178.12 (1.85 ; 354.4) | 0.048* | 0.051 |
| adiponectin | -382.56 (-768.92 ; 3.81) | 0.052 | *0.002* |
| leptin | 71.93 (-18.61 ; 162.46) | 0.119 | *0.000* |
| alpha_sma | -1.93 (-3.83 ; -0.02) | 0.047* | 0.351 |

| **Variable** | **Beta (DM*Sex)** | **p-value (DM*Sex)** | **p-value White** |
| --- | --- | --- | --- |
| log(ccl_2) | -0.91 (0.4 ; 0.22) | 0.004** | 0.596 |
| log(cd44s) | -0.62 (0.54 ; 0.35) | 0.004** | 0.318 |
| log(adiponectin) | -0.78 (0.46 ; 0.25) | 0.012* | 0.239 |
| log(leptin) | 0.04 (1.04 ; 0.7) | 0.845 | 0.051 |
