# Supplementary material for: Influence of diabetes mellitus on the pathological profile of aortic stenosis: a sex-based approach
Source: Cardiovasc Diabetol. 2023 Oct 17;22:280. doi: 10.1186/s12933-023-02009-w (PMC10583330; doi:10.1186/s12933-023-02009-w)
Supplement: Supplementary file 3 — Additional file 3: Figure S2. Effect interaction plots between the presence of diabetes and sex for pathological markers of AS in AVs from AS patients [file 12933_2023_2009_MOESM3_ESM.pptx]

## Slide 1
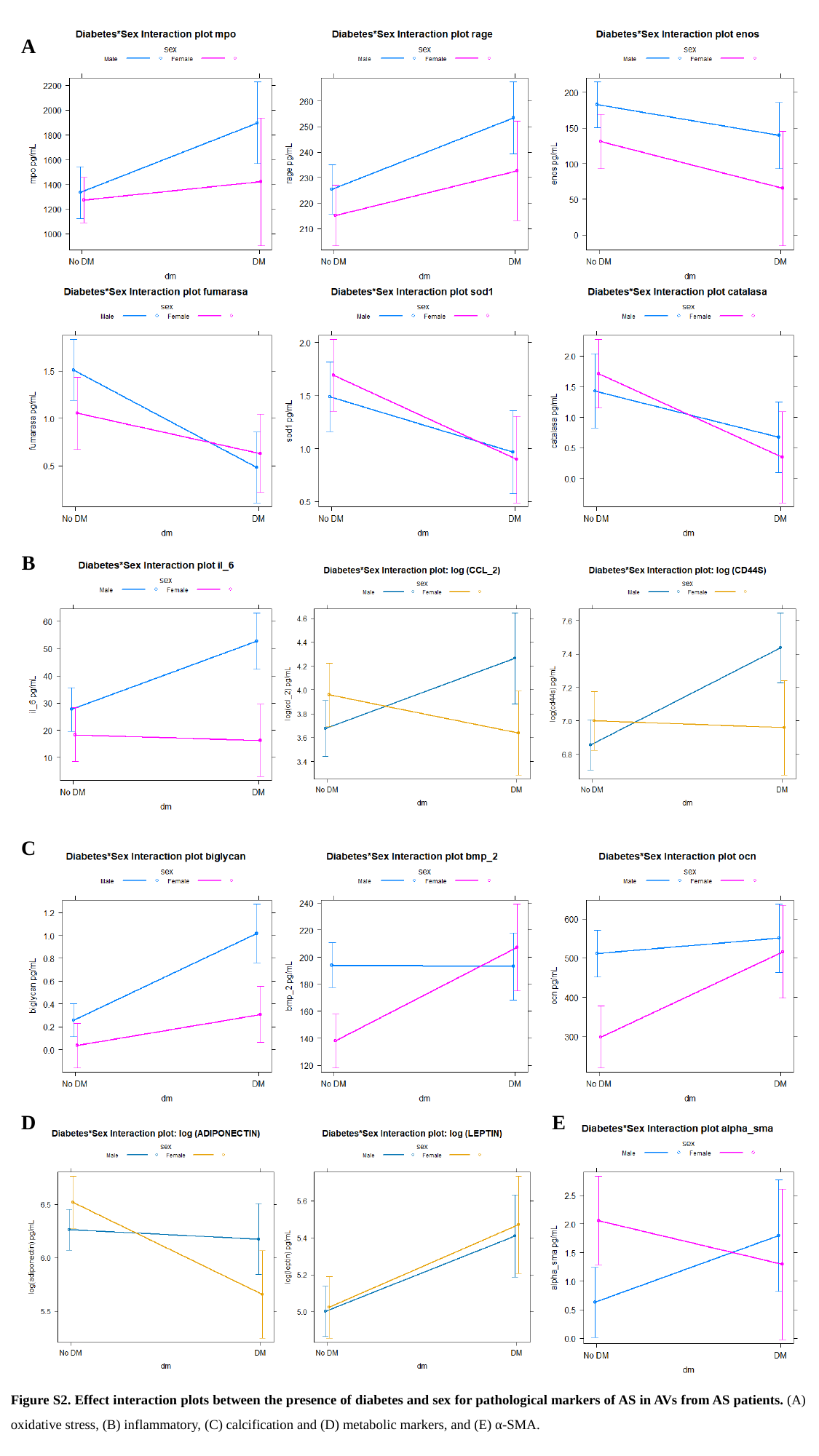

A
B
C
D
E
Figure S2. Effect interaction plots between the presence of diabetes and sex for pathological markers of AS in AVs from AS patients. (A) oxidative stress, (B) inflammatory, (C) calcification and (D) metabolic markers, and (E) α-SMA.
