# Supplementary material for: Influence of diabetes mellitus on the pathological profile of aortic stenosis: a sex-based approach
Source: Cardiovasc Diabetol. 2023 Oct 17;22:280. doi: 10.1186/s12933-023-02009-w (PMC10583330; doi:10.1186/s12933-023-02009-w)
Supplement: Supplementary file 4 — Additional file 4: Figure S3. Expression of oxidative stress, calcification, inflammatory and metabolic markers in non-diabetic VICs exposed to high-glucose levels [file 12933_2023_2009_MOESM4_ESM.docx]

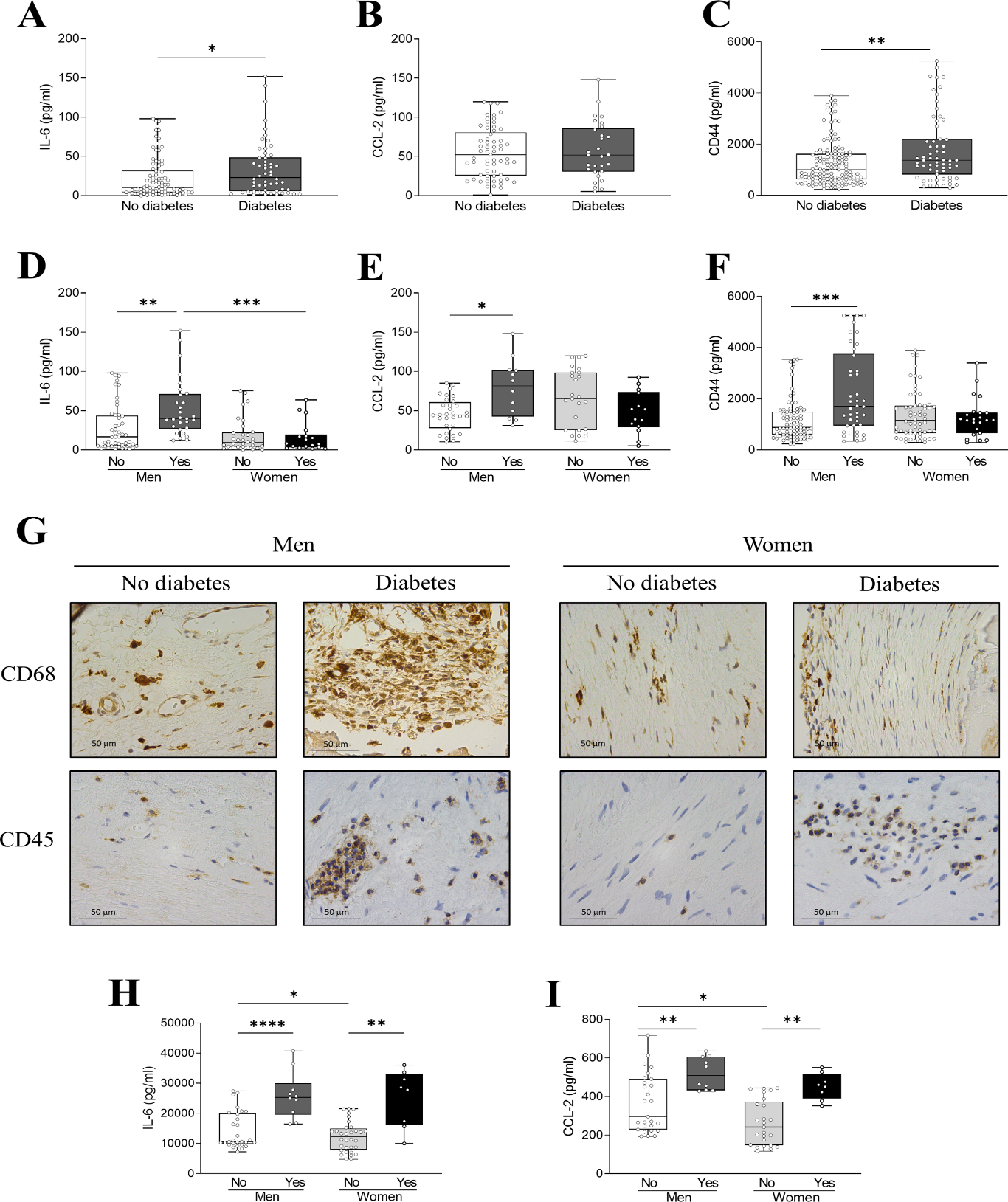


**Figure S3. Expression of oxidative stress, calcification, inflammatory and metabolic markers in non-diabetic VICs exposed to high-glucose levels.** MPO (**A**), RAGE (**B**), IL-6 (**C**), CCL-2 (**D**), BMP-4 (**E**), periostin (**F**), adiponectin (**G**) and IGFBP2 (**H**) protein levels in cell supernatant of VICs derived from AVs of male and female non-diabetic AS patients cultured in high glucose (4.5 g/L) conditions measured by ELISA. Time points assessed were 0, 24, 48 and 72 h. MPO, myeloperoxidase; RAGE, receptor for advanced glycation end-products; IL, interleukin; CCL, C-C motif chemokine ligand; BMP, bone morphogenetic protein; IGFBP, insulin like growth factor binding protein. Number of biological replicates: 4 men and 4 women; number of technical replicates: 6. **p* < 0.05, ***p* < 0.01, ****p* < 0.001, *****p* < 0.001
